# Associations of health status and diabetes among First Nations Peoples living on-reserve in Canada

**DOI:** 10.17269/s41997-021-00488-6

**Published:** 2021-06-28

**Authors:** Malek Batal, Hing Man Chan, Karen Fediuk, Amy Ing, Peter Berti, Tonio Sadik, Louise Johnson-Down

**Affiliations:** 1 Département de nutrition, Faculté de Médecine, Université de Montréal, Pavillon Liliane de Stewart, CP 6128 succ. Centre-Ville, Montréal, QC H3T 1A8 Canada; 2 Centre de recherche en santé publique de l’Université de Montréal et du CIUSS du Centre-sud-de-l’Île-de-Montréal (CReSP), 7101 Avenue du Parc, Montréal, QC H3N 1X7 Canada; 3 Department of Biology, University of Ottawa, 30 Marie Curie, Ottawa, ON K1N 6N5 Canada; 4 First Nations Food, Nutrition and Environment Study, University of Ottawa, 30 Marie Curie, Ottawa, ON K1N 6N5 Canada; 5 HealthBridge Foundation of Canada, 1 Nicholas Street, Suite 1004, Ottawa, ON K1N 7B7 Canada; 6 Assembly of First Nations, 55 Metcalfe Street, Suite 1600, Ottawa, ON K1P 6L5 Canada

**Keywords:** Indigenous, First Nations, Population health, Diabetes, Obesity, Autochtones, Premières Nations, Santé de la population, Diabète, Obésité

## Abstract

**Objective:**

Our objective is to describe self-reported health status, prevalence of diabetes and obesity and their associations in participants from the First Nations Food, Nutrition and Environment Study (FNFNES) in order to identify possible correlates of health in First Nations adults.

**Methods:**

FNFNES is a participatory study with First Nations Peoples living on reserve lands south of the 60^th^ parallel. Health and diabetes were self-reported, and prevalence of obesity was evaluated. Socio-demographic and lifestyle factors and traditional food (TF) activities were investigated for associations with health parameters.

**Results:**

High prevalence rates of overweight/obesity (78–91%) and diabetes (19% age-standardized prevalence) were found. Smoking rates were high and physical activity was low. In multivariable analyses, obesity was associated with region, income source, age, gender, smoking and self-reported health; diabetes and lesser self-reported health were associated with obesity and lower education. Diabetes was strongly associated with lesser self-reported health and weakly associated with being a smoker.

**Conclusion:**

We have identified possible correlates of health in this population that can help to better understand the underlying concerns and identify solutions for First Nations and their partners. We urge governments and First Nations to address the systemic problems identified with a holistic ecosystem approach that takes into consideration the financial and physical access to food, particularly TF, and the facilitation of improved health behaviour. New mechanisms co-developed with First Nations leadership should focus on supporting sustainable, culturally safe and healthy lifestyles and closing the gaps in nutrition and food insecurity.

## Résumé

**Objectifs:**

Décrire l’état de santé autodéclaré, les prévalences de diabète et d’obésité et leurs associations chez les participants de l’Étude sur l’alimentation, la nutrition et l’environnement des Premières Nations (EANEPN) afin d’identifier d’éventuelles associations avec la santé chez les adultes des Premières Nations.

**Méthode:**

L’EANEPN est une étude participative entreprise avec les Peuples des Premières Nations vivant dans les réserves au sud du 60^e^ parallèle. L’état de santé et le diabète ont été autodéclarés et la prévalence de l’obésité a été évaluée. Les facteurs sociodémographiques et de style de vie et les activités d’apports en aliments traditionnels (AT) ont été étudiés pour leurs associations avec les paramètres de santé.

**Résultats:**

Une prévalence élevée de surpoids/obésité (78–91%) et de diabète (19% – taux normalisé selon l’âge) a été observée. Le taux de tabagisme était élevé et l’activité physique était faible. Dans les analyses multivariées, l’obésité était associée avec la région, la source de revenu, l’âge, le sexe, le tabagisme et l’état de santé autodéclaré; le diabète et l’état de santé faible autodéclarés étaient positivement associés à l’obésité et à moins d’années d’éducation. Le diabète était fortement associé à un faible état de santé autodéclaré et légèrement avec le tabagisme.

**Conclusion:**

Nous avons identifié des corrélats de santé potentiels dans cette population qui peuvent nous aider à mieux comprendre les préoccupations sous-jacentes. Nous exhortons les gouvernements et les Premières Nations à s’attaquer de concert aux problèmes systémiques identifiés avec une approche écosystémique holistique qui prend en considération l’accès financier et physique à la nourriture, en particulier les AT, et qui facilite une amélioration dans les comportements en santé. De nouveaux mécanismes élaborés conjointement avec les dirigeants des Premières Nations devraient viser à soutenir des modes de vie durables, culturellement sécuritaires et sains et à combler les lacunes en matière de nutrition et d’insécurité alimentaire.

## Introduction

The United Nations Declaration on the Rights of Indigenous Peoples includes the right to “the enjoyment of the highest attainable standard of physical and mental health” (United Nations 2007). The well-being of First Nations Peoples is determined by a broad range of factors that include the structural determinants of health that are defined proximally to distally (Reading 2018).

First Nations individuals are more likely to report lower incomes, more single-parent households and lower education attainment, to experience higher rates of food insecurity, and to live in inadequate housing conditions as compared with non-Indigenous Canadians (Adelson 2005; Batal et al. 2021a; Chief Public Health Officer 2016). These inequities are rooted in historic and contemporary colonial policies that have led to a loss of cultural continuity and jurisdiction over traditional territories (Adelson 2005; Reading 2018; Reading and Wien 2009). These and the ongoing intergenerational trauma from the residential school system and other assimilation efforts contribute to poorer health outcomes in First Nations Peoples (Egeland and Harrison 2013; Public Health Agency of Canada and Pan-Canadian Public Health Network 2018; Reading and Wien 2009).

Indigenous views of health are holistic and seen as a balance among physical, mental, emotional, and spiritual well-being (King et al. 2009). The First Nations Regional Health Survey (FNRHS) reports that 44% of participants rate their health as very good or excellent (First Nations Information Governance Centre 2018b; Gionet and Roshanafshar 2013). Despite this, First Nations Peoples encounter major health disparities as compared with non-Indigenous populations (Adelson 2005; Chief Public Health Officer 2016; Gracey and King 2009; King et al. 2009). The life expectancy of First Nations individuals at birth is 5 years below that of other residents of Canada (Anderson et al. 2016; Statistics Canada 2017c). First Nations adults face a higher prevalence of obesity, chronic diseases such as diabetes, cardiovascular disease, cancer, and infectious diseases like tuberculosis, as well as mental health problems, as suggested by suicide rates (Chief Public Health Officer 2016; First Nations Information Governance Centre 2018a; Park et al. 2015; Statistics Canada 2016). Frohlich et al. (2006) argue that health disparities may be proxies for inequities in material resources and opportunities affecting First Nations communities.

Ecological changes from the degradation of the environment from industrial development and natural resource extraction activities and the concomitant climate change further threaten the ability of Indigenous Peoples to maintain traditional lifeways such as reliance on their traditional food (TF) systems (Ford et al. 2010; Ford 2012; Hectors et al. 2011; Lee et al. 2011; Li et al. 2006; Marushka et al. 2017a; Tam et al. 2013). Additionally, the chemical contamination of food and water from industrialization is linked to major health problems such as cancer and diabetes (Hectors et al. 2011; Lee et al. 2011; Li et al. 2006). Indigenous Peoples report feelings of grief related to the negative impact of climate-related changes to their lands, defined as ‘ecological grief’ (Cunsolo and Ellis 2018). Industrial development may contribute to this grief and the accompanying mental health concerns resulting from these ecological losses and their impact on traditional lifestyles (Cunsolo and Ellis 2018). Understanding the processes underlying this grief could help support the resourcefulness of individuals and communities facing these challenges.

Western world assessments do not consider Indigenous views of wellness or the resilience that these Peoples have shown to the myriad challenges they face (King et al. 2009). First Nations individuals face concerns over overweight/obesity, physical inactivity, alcohol use, diet and smoking (Betancourt et al. 2014). The prevalence of overweight/obesity in First Nations individuals has reached epidemic proportions (76%), and is consistently 16% higher than in other Canadians (60%) (First Nations Information Governance Centre 2012; Gionet and Roshanafshar 2013; Statistics Canada 2020b). First Nations individuals are less likely to report leisure physical activity than other Canadians (Chief Public Health Officer 2016; First Nations Information Governance Centre 2018b) and are more likely to smoke (Bruce et al. 2014; Chief Public Health Officer 2016; First Nations Information Governance Centre 2018b). Food insecurity, poor quality diet, and a decreasing proportion of TF have long been associated with nutrition-related health problems in First Nations Peoples (Kuhnlein and Receveur 1996; Lavigne-Robichaud et al. 2018; Power 2007; Willows 2005).

Our objectives are 1) to describe weight and self-reported health status, and prevalence of diabetes and their associations in participants in the First Nations Food, Nutrition and Environment Study (FNFNES); and 2) to identify possible cultural, lifestyle, socio-demographic or economic correlates of obesity, self-reported health and diabetes in First Nations adults. Issues relating to contaminants and their impact on health are reported elsewhere (Chan et al. 2021b; Marushka et al. 2021).

## Methods

The FNFNES is a participatory Canadian study of First Nations adults living south of the 60^th^ parallel. Sampling was conducted in multiple stages and is described elsewhere (Chan et al. 2021a). First Nations principles of Ownership, Control, Access and Possession (OCAP®) were respected and informed consent was requested from all individuals (Chan et al. 2021a; Schnarch 2004). All results were presented to the leadership and the members of participating First Nations communities in the regions and feedback was sought on the representativeness of the findings and incorporated before any results were disseminated in anonymized reports and articles. First Nations leadership and members had access to their Nation’s data while all publications were based on aggregate data to ensure anonymity.

A social/health/lifestyle questionnaire administered by First Nations interviewers collected data on: the main source of income (wages, social assistance, pensions, workers compensation/employment insurance or other sources), age groups (19–30 years, 31–50 years, 51–70 years, ≥71 years), the individual’s attained education (≤8 years, 9–12 years, ≥13 years), gender, diabetes and smoking. Participants were also asked about the employment status of household members along with a series of health-related questions in order to understand the relationships among diet, lifestyle and health risks. Participants were asked to identify their self-reported health on a 5-point scale: poor, fair, good, very good or excellent. Participants described their usual activities, classified as follows: ‘sitting and not walking very much’ (sedentary); ‘standing and walking around quite a lot but not carrying or lifting things’ (somewhat active); ‘often lifting light loads, climbing stairs or walking uphill’ (moderately active); or ‘doing heavy work or carrying heavy loads’ (highly active).

Questionnaires included household TF activity (at least one of fishing, hunting, collecting seafood, collecting plants/berries, or planting a garden) that was used as a proxy measure for TF intake (Batal et al. 2021b). Weekly market food costs for a family were calculated using the National Nutritious Food Basket, a tool that calculates the amount and cost of 64 basic food items intended to represent a basic nutritious diet for people of different ages and sexes (Batal et al. 2021a; Health Canada 2009). Distance to the nearest service centre and road access were determined using a remoteness indicator created by Indigenous and Northern Affairs Canada (Alasia et al. 2017).

Height and weight measurements were self-reported by 5328 individuals and measured in a subsample of 3584 individuals who agreed to have these values recorded. Lightly clothed participants’ weights were measured using a Seca 803 digital scale (Seca Systems and Scales, Hanover, MD, USA). Height of participants (shoeless) was assessed with a measuring tape on a level surface. Body mass index (BMI) was calculated using both measured and self-reported heights and weights by dividing the weight (in kilograms) by the square of the height (in metres). This index was used to classify participants into normal weight (18.5–24.9), overweight (25–29.9), obese class I (30–34.9), obese class II (35–39.9) and obese class III (40 and over). Measured and reported heights and weights were compared for individuals with both data in each region by gender to estimate bias. BMI values that were calculated with reported height and/or weight values were adjusted for bias in reporting by applying results from simple regression analyses by gender, using the observed height and weight measurements and comparing with the reported ones following the reduced model 4 as described by Gorber et al. (2008).

The FNFNES captured information on diabetes starting only in the second year of data collection in British Columbia and then in all subsequent regions. For this reason, all diabetes analyses had a lower sample size (*n* = 5028). Because the mean age of First Nations individuals is lower than that of the general Canadian population (Statistics Canada 2017a), age-standardized diabetes prevalence was calculated using the 1991 Canadian census data (Statistics Canada’s standard for vital statistics due to its relatively current population structure). Age standardization also allowed for comparison of populations with different age profiles (Statistics Canada 2017b).

Bivariate analyses of socio-demographic and lifestyle characteristics, obesity, self-reported health and diabetes were performed. These last guided the inclusion of independent variables in multivariable logistic regressions where the dependent variables were obesity, self-reported health, and diabetes. Our intent was to describe patterns of obesity, diabetes, and self-reported health (self-reported health was dichotomized as good (combined ‘excellent’ and ‘very good’ and ‘good’) and poor (combined ‘fair’ and ‘poor’)). The following independent variables were used in the model: Assembly of First Nations regions (British Columbia, Alberta, Saskatchewan, Manitoba, Ontario, Quebec and Labrador, and Atlantic (Newfoundland, New Brunswick, Nova Scotia, Prince Edward Island)); year-round road access to the community; number of individuals in the household working full-time (0, 1, or 2 or more); household TF activities (reported at least one of fishing, hunting, collecting seafood, collecting plants/berries, or planting a garden); main source of income (wages, social assistance, workers compensation or employment insurance, pension or other sources); age group (19–30, 31–50, 51–70, and ≥71 years); number of years of education (≤8 years, 9–12 years, and ≥13 years); gender; BMI category (normal, overweight, obese); smoking (yes/no); self-reported health (good (combined ‘excellent’ and ‘very good’ and ‘good’) and poor (combined ‘fair’ and ‘poor’)); and diabetes (yes/no) (Chan et al. 2021a). The continuous variables of household size and food basket cost were also included as predictors for self-reported health. Odds ratios were adjusted for all other variables.

Epi Info 3.5.4 was used to enter data (Centers for Disease Control and Prevention, Atlanta, GA, USA, 1988). SAS/STAT version 9.4 was utilized for data analysis (SAS, Cary, NC, USA, 2013). First Nations communities and household weights were applied for non-response and to ensure they represented the population. Population changes from 2008 to 2017 were also considered in the weighting. A ratio of populations was calculated by dividing the 2017 population by the reference-year population used. Year-end population data were obtained from Indigenous and Northern Affairs Canada Indian Registry System for 2017 (Government of Canada 2017). Adjustment factors were calculated individually for each community or band to ensure continued representativeness of the data to First Nations Peoples in each region. The balanced repeated replication (BRR) option for variance estimation was employed in the multivariable analysis (‘VARMETHOD=BRR’) using PROC SURVEYLOGISTIC in SAS randomly generating 500 replicate weights using Bootstrap subsamples of the full sample.

## Results

**Fig. 1:**
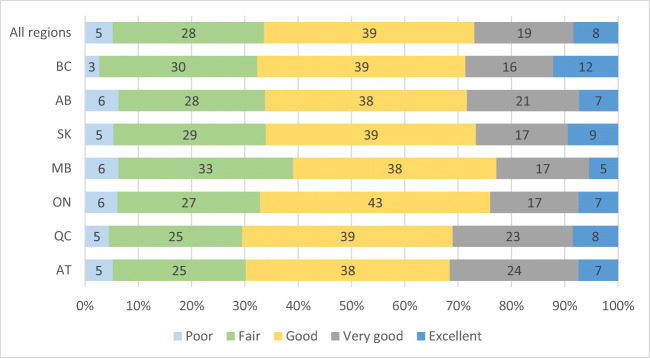
Measures of self-reported health status reported by First Nations participants in Canada from the First Nations Food, Nutrition and Environment Study (FNFNES) 2008–2018

**Fig. 2:**
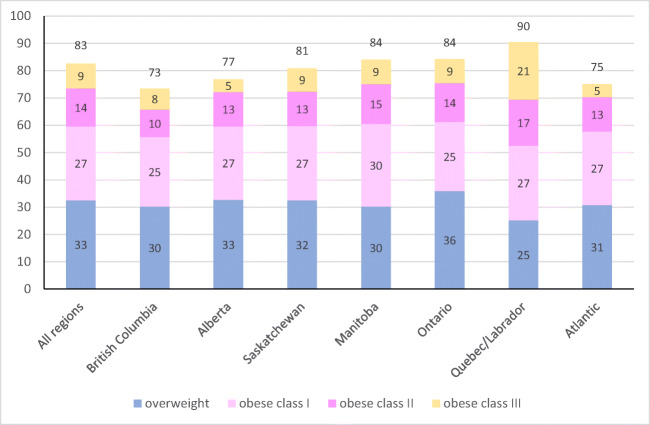
Proportion of First Nations adults who are overweight or obese in Canada from the FNFNES, 2008–2018. Overweight classified as BMI 25–29.9, obese class I as 30–34.9, obese class II as 35–39.9, and obese class III as 40 or greater

**Fig. 3:**
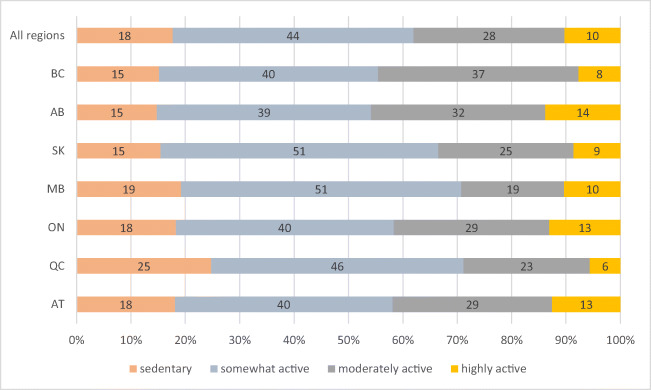
Proportion of self-reported individual physical activity levels among First Nations participants in Canada from the FNFNES, 2008–2018

Sample size where data were complete for health, diabetes, and BMI was 5023 (because diabetes prevalence was not measured in the first year of the study) and 5328 for analyses excluding diabetes (Table 1). Twenty-seven percent of participants reported ‘excellent’ or ‘very good’ health, 39% ‘good’, and 33% ‘fair’ or ‘poor’ health (Table 1, Fig. 1). Eighty-two percent of all adults were considered overweight or obese and results ranged from 78% to 91% in all regions of Canada (Table 1, Fig. 2). Class III obesity in Quebec was more than double that of any other region (Fig. 2). Approximately two thirds of all adults (62%) were classified as ‘sedentary’ or ‘somewhat active’ (Table 1, Fig. 3). At the regional level, the rate of moderate and high physical activity appeared highest in Alberta (46%) and lowest in Manitoba (39%) (Fig. 3). Over half (52%) of First Nations adults reported that they smoked cigarettes (Table 1) and smoking prevalence was lowest in British Columbia (39%).

**Table 1:** Characteristics of First Nations participants in Canada from the First Nations Food, Nutrition and Environment Study (FNFNES) 2008–2018

Characteristic^a^	All % [95% CI]	Women % [95% CI]	Men % [95% CI]
*n*	5328	3395	1933
Women	65.5 [61.2, 69.7]		
Men	34.5 [30.3, 38.8]		
Age (mean (SE))	44.9 (0.58)	45.0 (0.57)	44.7 (0.85)
19–30 years	18.4 [16.1, 20.7]	17.4 [15.2, 19.6]	20.3 [16.4, 24.2]
31–50 years	47.3 [45.2, 49.4]	47.6 [44.7, 50.5]	46.7 [42.5, 50.9]
51–70 years	29.3 [6.9, 31.7]	30.3 [27.3, 33.3]	27.5 [23.6, 31.5]
≥71 years	4.95 [3.97, 5.92]	4.68 [3.69, 5.67]	5.46 [3.80, 7.11]
Years of education (mean (SE))	10.7 (0.12)	11.0 (0.14)	10.3 (0.23)
≤8 years	20.2 [17.2, 32.2]	19.1 [15.3, 22.9]	22.2 [18.1, 26.4]
9–12 years	61.5 [58.5, 64.5]	60.2 [56.5, 64.0]	63.9 [60.0, 67.8]
≥13 years	18.3 [15.4, 21.3]	20.7 [17.5, 23.9]	13.8 [10.0, 17.6]
Household size (mean (SE))	4.78 (0.11)	4.95 (0.14)	4.46 (0.17)
Number of individuals working full-time in the household	0.98 (0.05)	1.00 (0.05)	0.94 (0.06)
2 or more	29.3 [24.6, 33.9]	30.4 [24.8, 35.8]	27.2 [21.6, 32.8]
1	31.5 [29.8, 33.2]	31.3 [28.8, 33.8]	31.8 [28.2, 35.4]
0	39.3 [35.1, 43.4]	38.3 [34.3, 42.4]	41.0 [35.0, 47.0]
Income source			
Wages	51.8 [45.2, 57.4]	52.3 [46.4, 58.3]	50.9 [44.5, 57.3]
Social assistance	28.6 [24.4, 32.8]	28.0 [23.5, 32.6]	29.7 [243.7, 34.7]
Workers compensation/employment insurance	5.57 [3.48, 7.65]	5.53 [2.70, 8.37]	5.63 [4.06, 7.20]
Pension/senior’s benefit^b^	10.4 [9.02, 11.8]	10.2 [8.97, 11.5]	10.8 [8.15, 13.4]
Other	3.55 [1.57, 5.53]	3.85 [1.51, 6.19]	2.97 [1.48, 4.46]
Body mass index (mean (SE))	30.9 (0.26)	31.3 (0.21)	30.1 (0.45)
Normal weight	17.6 [15.6, 19.6]	17.5 [15.4, 19.6]	17.9 [14.8, 21.1]
Overweight	32.6 [30.5, 34.7]	29.9 [28.0, 31.8]	37.7 [34.0, 41.5]
Obese	49.8 [47.0, 52.6]	52.6 [50.3, 55.0]	44.3 [39.2, 49.4]
Diabetes^c^	21.4 [18.8, 24.1]	21.7 [19.1, 24.4]	20.9 [16.3, 25.5]
Smoking	52.3 [48.9, 55.8]	52.8 [48.6, 57.0]	51.5 [47.0, 56.0]
Self-reported health^d^			
Very good	26.9 [24.6, 29.2]	25.3 [22.5, 28.2]	29.8 [26.6, 33.1]
Good	39.5 [37.4, 41.6]	39.7 [36.9, 42.5]	39.0 [35.3, 42.7 ]
Poor	33.6 [31.3, 36.0]	34.9 [32.1, 37.7]	31.1 [28.0, 34.3]
Physical activity			
Sedentary	17.6 [14.5, 20.7]	20.9 [17.0, 24.7]	11.5 [9.28, 13.7]
Somewhat active	44.3 [42.0, 46.7]	47.5 [43.1, 51.9]	38.3 [34.1, 42.4]
Moderately active	27.8 [24.8, 30.7]	26.0 [22.7, 29.3]	31.1 [26.9, 35.4]
Highly active	10.3 [8.78, 11.7]	5.58 [4.19, 6.97]	19.1 [16.4, 21.9]
Year-round road	91.7 [87.8, 957]	92.1 [88.4, 958]	91.0 [86.1, 95.8]
Household traditional food activities^e^ (mean (SE))	67.8 [63.8, 71.8]	65.3 [61.0, 69.6]	72.6 [68.1, 77.0]
Food basket cost (mean (SE))	$215.41 ($3.91)	$215.97 ($3.79)	$214.34 ($4.87)

*CI* confidence interval, *SE* standard error of the mean

^a^Weighted data

^b^Missing values for first year of data collected on BC removed from analysis

^c^Self-reported diabetes

^d^Good self-reported health included reports of ‘excellent’, ‘very good’ and ‘good’; poor self-reported health included reports of ‘fair’ and ‘poor’

^e^Household traditional food activities defined as reporting at least one of fishing, hunting, collecting seafood, collecting plants/berries, or planting a garden

**Fig. 4:**
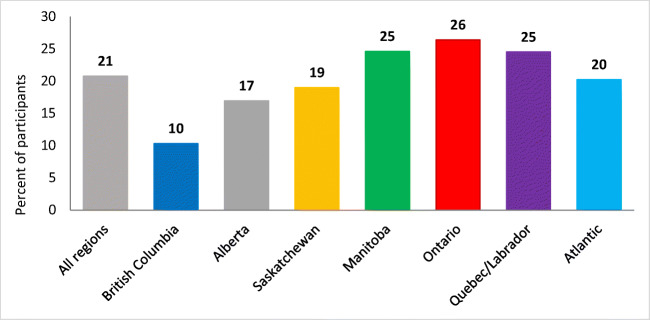
Crude weighted prevalence of diabetes in First Nations participants in Canada from the FNFNES, 2008–2018

The crude weighted, self-reported prevalence of diabetes among First Nations adults was 21% (Fig. 4) while the age-standardized prevalence was 19% (21% for women and 17% for men). The lowest prevalence was 10% in British Columbia with that in other regions ranging from 17% to 26% (Fig. 4). Only 8% of adults under the age of 40 reported having diabetes as compared with 29% for those older than 40. Most adults who reported having diabetes indicated it was type 2 diabetes although 22% did not know what type they had. Overall, 45% of adults with diabetes reported that they smoked. There seemed to be some regional variation, with the lowest rate of smoking among adults with diabetes in the Atlantic region and the highest in Ontario.

In bivariate analyses, younger individuals, men, smokers, those who reported good to excellent health, those who reported any physical activity, and non-diabetics were less likely to be obese (Table 2). Diabetes was less prevalent in British Columbia than in Alberta, Manitoba, Ontario and the Atlantic region. It was more prevalent in individuals over 50 years of age, in overweight/obese individuals, in those with 8 years or less of education, in sedentary individuals, and in those who reported poor health (Table 2). Good self-reported health was reported more when 2 or more individuals worked full-time in the household vs. in households with no full-time workers, in individuals 50 years of age or less vs. those 51–70 years of age, in those who were not obese, in those with more than 8 years of education, and in those reporting more physical activity (Table 2).

**Table 2:** Associations of obesity, diabetes and self-reported health with selected socio-demographic and lifestyle characteristics of First Nations participants in Canada from the FNFNES, 2008–2018

		Obesity^a^ (%)	95% CI		Diabetes^b**,**c^ (%)	95% CI		Good self-reported health^d^ (%)	95% CI	
			LL	UL		LL	UL		LL	UL
Region	British Columbia	43.2	33.3	53.2	10.3	7.27	13.4	67.6	59.9	75.4
	Alberta	44.2	35.8	52.6	17.3	14.1	22.5	66.2	59.9	72.5
	Saskatchewan	48.4	43.8	59.6	20.3	13.4	37.2	66.0	63.3	68.8
	Manitoba	53.8	48.1	59.6	24.8	19.0	30.7	60.9	57.2	64.6
	Ontario^c^	48.2	43.8	52.6	27.	21.8	33.5	67.1	62.2	71.9
	Quebec/Labrador	65.3	56.3	74.3	23.9	12.5	35.4	70.5	60.9	80.0
	Atlantic region	48.4	45.1	51.8	20.2	17.3	23.2	69.8	65.8	73.9
Year-round road	No	50.5	43.6	57.3	23.9	17.2	30.5	69.6	64.2	74.9
	Yes	49.8	46.8	52.8	21.2	18.4	24.0	66.0	63.5	68.6
Number of individuals working full-time in the household	2 or more	46.5	42.9	50.1	18.3	14.9	21.7	69.4	65.7	73.0
	1	51.7	47.4	55.9	20.1	15.9	24.2	69.4	65.0	73.8
	0	52.4	47.8	57.0	24.7	21.3	28.2	61.7	58.2	65.2
Household traditional food activities^e^	Yes	50.3	47.3	53.4	21.1	18.3	23.9	68.6	65.8	71.6
	No	48.8	44.3	53.2	22.0	18.6	25.4	61.3	56.6	66.0
Income source	Wages	52.5	48.4	56.6	17.8	15.2	20.4	71.1	68.5	73.7
	Social assistance	43.6	39.1	48.2	19.3	16.1	22.5	61.9	56.9	66.8
	Workers compensation/employment insurance	50.9	37.1	64.7	24.2	15.4	32.9	55.0	44.5	65.5
	Pension/senior’s benefit^c^	53.1	45.6	60.7	42.3	36.4	48.2	59.3	52.5	66.2
	Other	49.5	36.3	62.5	27.0	6.81	47.2	72.4	60.5	84.2
Age group	19–30 years	40.7	36.2	45.2	4.68	2.08	7.28	70.1	66.2	73.9
	31–50 years	49.6	45.4	53.7	16.0	12.2	19.9	69.2	66.6	71.8
	51–70 years	57.1	53.8	60.5	39.1	34.8	43.5	60.3	54.8	65.8
	≥71 years	43.5	34.0	53.0	35.0	28.0	42.0	60.7	49.6	71.9
Body mass index	Normal weight	NA			9.31	5.80	12.8	68.4	63.5	73.3
	Overweight	NA			24.0	21.0	26.9	74.0	69.8	78.3
	Obese	NA			28.8	25.9	31.8	60.6	57.7	63.5
Years of education	≤8 years	53.7	48.4	58.9	34.7	30.8	38.6	55.6	49.5	61.8
	9–12 years	48.3	45.0	51.5	17.9	15.0	20.8	67.6	64.1	71.2
	≥13 years	50.9	46.6	55.3	18.7	15.0	22.4	73.8	68.6	79.0
Gender	Men	44.3	39.2	49.4	20.9	16.3	25.4	68.9	65.8	72.1
	Women	52.8	50.4	55.2	21.7	19.1	24.3	65.0	62.2	67.8
Smoking	No	54.6	50.5	58.7	25.2	21.4	29.1	66.9	62.4	71.3
	Yes	45.5	42.5	48.6	18.1	15.2	21.0	65.9	63.8	67.9
Physical activity	Sedentary	61.2	57.7	64.7	33.4	27.0	39.7	46.9	40.4	53.5
	Somewhat active	49.7	44.9	54.4	21.2	18.8	23.5	66.1	64.2	68.1
	Moderately active	45.4	41.4	49.4	16.2	11.9	20.5	76.0	71.7	80.3
	Highly active	42.2	35.0	49.4	16.2	10.1	22.2	74.7	68.8	80.7
Self-reported health	Very good	39.9	33.5	46.3	12.2	9.81	14.5	NA		
	Good	49.4	45.3	53.4	18.4	14.6	22.2	NA		
	Poor	58.3	54.6	62.0	32.5	28.9	36.0	NA		
Diabetes	Yes	67.5	62.4	75.6	NA			NA		
	No	45.4	42.1	48.7	NA			NA		

*CI* confidence interval, *LL* lower limit, *UL* upper limit, *NA* not applicable

^a^BMI≥30

^b^Self-reported diabetes

^c^Missing values for first year of data collected on BC removed from analysis

^d^Good self-reported health included reports of ‘excellent’, ‘very good’ and ‘good’; poor self-reported health included reports of ‘fair’ and ‘poor’

^e^Household traditional food activities defined as reporting at least one of fishing, hunting, collecting seafood, collecting plants/berries, or planting a garden

In multivariable analyses, obesity was more likely in Quebec/Labrador than in Ontario and British Columbia, in those on social assistance vs. those on pension/senior’s benefits, in individuals 31–70 years of age vs. 71 years or older, in men, in non-smokers, in sedentary individuals, in those with poor self-reported health, and in those who were diabetic (Table 3). In these adjusted analyses, likelihood of reporting diabetes was lower among participants who were in British Columbia vs. Ontario and Manitoba, who were younger (19–50 years vs. 71 years and older), who were not obese, who were somewhat or moderately active, or who reported ‘good’ to ‘excellent’ health (Table 3). The odds of reporting poor health varied by region (Atlantic region vs. Ontario), obesity, education, physical activity, and the diabetic status of participants (Table 3).

**Table 3:** Adjusted odds ratio for obesity, diabetes and self-reported health of selected socio-demographic and lifestyle characteristics of First Nations participants in Canada from the FNFNES, 2008–2018

		Obesity^a^ (AOR^b^)	95% CI		Diabetes^c**,**d^ (AOR^b^)	95% CI		Self-reported health^e^ (AOR^b,f^)	95% CI	
			LL	UL		LL	UL		LL	UL
Region	British Columbia	0.74	0.47	1.18	0.29^***^	0.17	0.49	0.89	0.50	1.58
	Alberta	0.92	0.59	1.43	0.66	0.38	1.14	0.96	0.62	1.48
	Saskatchewan	0.97	0.66	1.42	0.78	0.42	1.45	0.88	0.60	1.29
	Manitoba	1.22	0.87	1.72	0.87	0.49	1.54	0.96	0.62	1.48
	Ontario	Ref.			Ref.			Ref.		
	Quebec/Labrador	1.88^***^	1.18	3.00	0.78	0.42	1.44	0.63^**^	0.42	0.95
	Atlantic region	1.03	0.78	1.36	0.86	0.57	1.32	0.76	0.52	1.12
Year-round road	No	Ref.			Ref.			0.70	0.41	1.18
	Yes	0.64	0.33	1.26	1.37	0.65	2.91	Ref.		
Number of individuals working full-time in the household	2 or more	1.05	0.71	1.56	0.96	0.60	1.54	1.04	0.69	1.57
	1	1.15	0.87	1.50	1.01	0.73	1.39	1.00	0.69	1.46
	0	Ref.			Ref.			Ref.		
Household traditional food activities^g^	Yes	1.10	0.89	1.36	1.15	0.92	1.43	0.84	0.66	1.07
	No	Ref.			Ref.			Ref.		
Income source	Wages	0.88	0.55	1.42	0.58^*^	0.34	1.00	0.89	0.58	1.36
	Social assistance	0.66^**^	0.45	0.97	0.73	0.49	1.09	1.19	0.78	1.82
	Workers compensation/employment insurance	0.82	0.37	1.85	0.77	0.36	1.67	1.71^*^	1.00	2.93
	Pension/senior’s benefit	Ref.			Ref.			Ref.		
	Other	0.72	0.40	1.30	1.31	0.38	4.49	0.72	0.32	1.62
Age group	19–30 years	1.30	0.81	2.09	0.16^***^	0.05	0.48	0.96	0.61	1.50
	31–50 years	1.70^**^	1.03	2.81	0.63	0.32	1.25	1.00	0.63	1.57
	51–70 years	2.17^***^	1.40	3.35	1.88^*^	0.97	3.66	1.27	0.79	2.04
	≥71 years	Ref.			Ref.			Ref.		
Body mass index	Normal weight				0.33^***^	0.21	0.51	0.74^**^	0.55	0.99
	Overweight				0.54^***^	0.39	0.75	0.58^***^	0.45	0.75
	Obese	Ref.			Ref.			Ref..		
Years of education	≤8 years	Ref.			Ref.			Ref.		
	9–12 years	0.91	0.71	1.18	0.72^*^	0.50	1.03	0.66^**^	0.44	1.00
	≥13 years	0.94	0.71	1.25	0.71^*^	0.47	1.06	0.52^***^	0.35	0.76
Gender	Men	Ref.			Ref.					
	Women	1.24^**^	1.02	1.51	0.89	0.63	1.27	1.01^*^	0.86	1.18
Smoking	No	1.43^***^	1.16	1.77	1.22^*^	0.96	1.54	0.92	0.71	1.19
	Yes	Ref.			Ref.			Ref.		
Physical activity	Sedentary	Ref.			Ref.			Ref.		
	Somewhat active	0.72^**^	0.54	0.94	0.70^**^	0.50	0.99	0.46^***^	0.34	0.63
	Moderately active	0.70^***^	0.55	0.89	0.64^**^	0.42	0.99	0.30^***^	0.21	0.43
	Highly active	0.64^*^	0.40	1.01	0.67	0.40	1.13	0.33^**^	0.20	0.56
Self-reported health	Very good	0.49^***^	0.36	0.67	0.35^***^	0.24	0.51	NA		
	Good	0.73^**^	0.56	0.94	0.55^***^	0.46	0.66	NA		
	Poor	Ref.			Ref.			NA		
Household size		1.04^*^	1.00	1.09	1.02	0.96	1.08	1.02	0.97	1.06
Food basket cost		1.00	1.00	1.01	1.00	0.99	1.00	1.00	1.00	1.00

*CI* confidence interval, *LL* lower limit, *UL* upper limit, *AOR* adjusted odds ratio, *NA* not applicable

^a^BMI≥30

^b^Odds ratio adjusted for all other variables in a multivariable logistic regression

^c^Self-reported diabetes

^d^Missing values for first year of data collected on BC removed from analysis

^e^Good self-reported health included reports of ‘excellent’, ‘very good’ and ‘good’; poor self-reported health included reports of ‘fair’ and ‘poor’

^f^Odds ratio of having ‘poor’ self-reported health

^g^Household traditional food activities defined as reporting at least one of fishing, hunting, collecting seafood, collecting plants/berries, or planting a garden

Different from referent in multivariable logistic regression * <0.1 ** <0.05 *** <0.01

## Discussion

Despite centuries of marginalization, First Nations have overcome many challenges affecting their well-being. As a result of inequities stemming from a long history of colonial practices beyond their control, First Nations Peoples face disturbing disparities in health and well-being as compared with others in Canada that result in poor diets, high rates of food insecurity, and suboptimal physical activity (Adelson 2005; Frohlich et al. 2006; Reading 2018). Researchers have also identified a process of ‘ecological grief’ affecting the well-being and traditional lifestyles of Indigenous Peoples stemming from colonization and the continuing destruction of habitats (Cunsolo and Ellis 2018). In a cohort analysis of First Nations adults, Park et al. (2015) report that diabetes, alcohol and drug use disorders, and unintentional injuries are the main contributors to excess avoidable deaths and that disparities in comparison with non-Indigenous Canadians are larger in men and younger individuals. Bruce et al. (2014) report more obesity, diabetes, and high blood pressure in off-reserve First Nations individuals as compared with their non-Indigenous counterparts.

BMI is a proxy measure of body fat, and overweight and obesity and measures of excess body weight are associated with increased risks of developing health problems such as diabetes and heart disease (Batal and Decelles 2019; Betancourt et al. 2014). In our study, First Nations individuals experience poor health compromised by a very high prevalence of obesity (double that of other Canadians) (Statistics Canada 2016) and that was associated with sedentary physical activity. Batal and Decelles (2019) found that on-reserve First Nations individuals have higher obesity than those living off-reserve. Results from the region of Quebec and Labrador appeared to indicate higher obesity, with more class III obesity, than in other regions; this may have resulted from the sample randomly including some First Nations communities with a greater prevalence of obesity.

Sixty-six percent of First Nations individuals reported that they were in good health, pointing to the resilience and strength with which First Nations individuals respond to chronic disease. Age-standardized prevalence of diabetes in this population was almost triple that of the 7.3% reported nationally in 2017 for Canadians aged 12 and older, but similar to that reported in the 2015/2016 FNRHS (First Nations Information Governance Centre 2018b; Statistics Canada 2018). Diabetes was less prevalent among adults reporting wages as compared with those on pension/senior’s benefits after adjusting for age and other variables. Lower income and poverty have long been associated with poorer health (Adelson 2005) and Park et al. (2015) found that a considerable share of the disparities in avoidable mortality result from inequities in education and income in First Nations adults living off-reserve. Notwithstanding the high prevalence across the country, prevalence of diabetes was lowest in British Columbia at 10% and further work to determine the reasons for this difference could help other regions. It is noteworthy that TF intake is the highest in British Columbia as compared with the other regions, as reported elsewhere (Batal et al. 2021b). Marushka et al. (2017b) also report an inverse association between traditional fish consumption and diabetes in a subsample of the same study, pointing to a potential protective effect of TF against diabetes.

Twenty-seven percent of adults in the FNFNES reported their health was ‘excellent’ or ‘very good’, whereas in the FNRHS, 37.8% of First Nations adults nationally describe their health as ‘excellent’ or ‘very good’ (First Nations Information Governance Centre 2018b). It is difficult to conclude whether this striking difference is a time trend or a result of different methodology between the two studies as, unlike the FNFNES, the FNHRS includes First Nations communities above the 60^th^ parallel (First Nations Information Governance Centre 2018b). In contrast, in the general Canadian population, 61% of all Canadians aged 12 years and over in 2017/2018 report that their health is ‘very good’ or ‘excellent’ (Statistics Canada 2019). First Nations’ concept of ‘good’ health or wellness may not be captured by a question of this type as their views of health differ from those of other Canadians (King et al. 2009).

Lifestyle factors affecting health, such as smoking and physical activity, are of concern in this study. Smoking rates (52%) were similar to those reported for First Nations adults living on-reserve across Canada in the FNRHS (53%) (First Nations Information Governance Centre 2018a) and higher than those for other Canadians (15%) (Reid et al. 2019). Higher prevalence of smoking is known to increase the risk of cancer and cardiovascular disease and may be exacerbating the already high prevalence of these diseases in this population (Chief Public Health Officer 2016; Park et al. 2015; World Health Organization 2009).

Despite a long history of colonial practices that have tried to impose European values (Reading 2018), the cultures of First Nations have continued to thrive and be important to First Nations Peoples living in Canada, as evidenced by the persistence of TF acquisition activities. It has long been established that TF contribute to the good health and well-being of Indigenous Peoples (Batal et al. 2021b; Willows et al. 2019) and higher TF intake improves the well-being of First Nations individuals (First Nations Information Governance Centre 2018b). Physical activity is also associated with TF intake (First Nations Information Governance Centre 2018b) and has health benefits on its own.

While this is true, 64% of First Nations reported being sedentary or somewhat active as compared with 56% of First Nations in the FNRHS and 42% of all Canadians (First Nations Information Governance Centre 2018b; Statistics Canada 2019). Some of this difference may be attributable to differing methodology and the apparent low activity may not reflect a true Indigenous perspective.

Levels of environmental contaminants such as mercury and persistent organic pollutants are concerns in First Nations Peoples as these may be linked to health problems such as cancer and diabetes (Hectors et al. 2011; Lee et al. 2011; Li et al. 2006; Marushka et al. 2017a; Marushka et al. 2018). Contaminants are found in some of the TF prized by these peoples, but the complex nature of the effect of these on health was beyond the scope of this article and this has been addressed elsewhere (Chan et al. 2021b; Marushka et al. 2021).

When results were shared with individual First Nations communities and feedback was received about the pertinence of the research results to First Nations, First Nations leadership and members voiced tremendous concern with regard to issues of environmental contamination of water and TF. However, the high rates of obesity and diabetes did not seem to elicit an equivalent response almost systematically in all visited communities. While this research cannot identify the reason behind the absence of a strong reaction to what is considered a systemic health issue with roots in a compromised food system which undermines access to healthy TF (Batal et al. 2021b; Kuhnlein 2018; Kuhnlein and Receveur 1996; Kuhnlein et al. 2004; Sheikh et al. 2011), it is possible that individuals still consider obesity and diabetes as outcomes of personal choice and lifestyle (Browne et al. 2013) or that these health challenges come from a sense of powerlessness resulting from a changing way of life brought about by environmental dispossession (Richmond and Ross 2009).

It is however clear that these health issues are of a structural nature and need to be addressed in Indigenous-led, strength-based, culturally appropriate solutions that can be called upon to mitigate the health consequences of these diseases (Domingo et al. 2020), with a clear understanding of the weight of history and colonialism in their emergence (Frohlich et al. 2006; Hackett 2005). In a diabetes study conducted in Alberta, Oster et al. (2014) found a lower prevalence of diabetes in First Nations with more of what they termed “cultural continuity”, measured by traditional Indigenous language knowledge. Warbrick et al. (2019) in speaking of the issue of stigmatization of Mauri in New Zealand state “Blaming individuals, or one or more ethnic groups, diverts attention from policies which favour commercial interest over health, with weak restrictions on the sale and marketing of certain types of food, and is an easy sell when indigenous groups are perceived as ‘offenders’”.

### Strengths and limitations

The strengths of our study included a large representative sample of First Nations adults and results were weighted by community, household and individual as well as for population growth over the 8 years of data collection. First Nations researchers and leaders were involved in the planning and execution of the data collection. Community-based participatory research requires a large investment in social capital throughout and beyond the scope of the research mandate. The benefits of this include the possibility of more relevant research questions, increased data use and dissemination, and the potential to establish sustainable partnerships for project expansion or future research, all of which can lead to better policy and health outcomes. Input from community leaders was invaluable to the planning for this study and reports were presented to First Nations communities for comments before they were released. Generally speaking, results from this study confirmed First Nations’ perceptions about the health status in their respective Nations, with an emphasis on personal responsibility often voiced by participants, pointing to the need for better exploring with research participants the historic and environmental drivers of chronic disease in general, and obesity and diabetes in particular.

One of the limitations of our study was not capturing diabetes in the first year of data collection, and because these data were not included in our diabetes prevalence, analyses may have resulted in a less representative estimate of diabetes prevalence in British Columbia. Also, the overall prevalence of diabetes in our study may have been underestimated as reports indicate that undiagnosed cases exist as a result of the ever-increasing burden of this disease (Rosella et al. 2020; Young et al. 2000). Health can be measured in many ways: self-reported physical or mental health or disease prevalence. We chose to use health outcomes like obesity, diabetes, and health behaviours in our analyses (e.g., smoking and self-reported health) for the sake of comparability with standard questions used in Canadian data such as from the Canadian Community Health Survey (Statistics Canada 2020a). However, our question on self-reported health may not have properly addressed Indigenous concepts of well-being (King et al. 2009).

Since measures of health and prevalence of diabetes were self-reported, they were most likely influenced by age and culture as well as access to proper screening in communities (Jylha 2009; Mora et al. 2008). Because no direct measures of income were recorded, education, source of income, and number of individuals in the household working full-time were used as proxy measures of this. Food costs were only collected for a subset of foods purchased by this population (Batal et al. 2021a; Health Canada 2009). Last, we did not address the health impact of contaminants or diet (except TF activities as a proxy for TF intake), but these have been addressed elsewhere (Batal et al. 2021b; Batal et al. 2021c; Chan et al. 2021b; Marushka et al. 2021).

## Conclusion

As we continue to identify health challenges faced by First Nations Peoples through participatory research requested by First Nations themselves (Chan et al. 2021a) and strive to identify ways for improving health and wellness, it is important to recognize that First Nations Peoples’ self-determination is of critical importance. They continue to build on their strengths and overcome the attacks on their way of life from historical colonial policies that challenge the health of the environment and their Peoples and access to healthy food and lifestyle options. Beyond the possible correlates of health that we have identified in this population and that can lead to a better understanding of some of the underlying concerns, these many factors impacting the health of First Nations Peoples will require a concerted effort at all levels of governance, especially by First Nations Peoples themselves, provided that their agency is strengthened with adequate political and financial support. Additionally, policy and program development need to consider the importance and the critical influence of traditional knowledge and activities in shaping responses to health challenges (First Nations Information Governance Centre 2018b).

At the individual level, access to resources (money, equipment), knowledge, and environmental challenges have a strong influence on behaviours that affect the well-being of First Nations Peoples and the prevalence of diabetes and obesity. Because our understanding remains limited about the magnitude of the impact from factors beyond the control of individuals, including policies, governance and jurisdiction, location, access to appropriate education, housing, and culturally safe health services, as well as social networks on adults’ lifestyle and health, we need to continue monitoring the health situation of First Nations Peoples and report on progress.

Along with First Nations leadership, we urge governments and decision-makers to urgently address the systemic problems relating to health and the environment affecting First Nations Peoples. These initiatives should do so in a manner that supports First Nations-led leadership and solutions and that does not stigmatize and blame the individuals for their health status. Taking a holistic approach, this needs to take into consideration the financial and physical access to food, particularly traditional foods, and the facilitation of improved health behaviour. New mechanisms co-developed with First Nations leadership should focus on supporting sustainable and healthy lifestyles and closing the gaps in nutrition and food insecurity.

## Data Availability

Data are owned by each participating community. The Assembly of First Nations is data custodian and any requests will be addressed to AFN through the corresponding author.

## Declarations

### Code availability

Analysis codes can be shared upon reasonable request.
